# Engagement with fact-checked posts on Reddit

**DOI:** 10.1093/pnasnexus/pgad018

**Published:** 2023-01-27

**Authors:** Robert M Bond, R Kelly Garrett

**Affiliations:** School of Communication, Ohio State University, 154 N. Oval Mall, Columbus, OH 43210, USA

**Keywords:** contested news, social media, engagement, fact-checks

## Abstract

Contested factual claims shared online are of increasing interest to scholars and the public. Characterizing temporal patterns of sharing and engagement with such information, as well as the effect of sharing associated fact-checks, can help us understand the online political news environment more fully. Here, we investigate differential engagement with fact-checked posts shared online via Reddit from 2016 to 2018. The data comprise ∼29,000 conversations, ∼849,000 users, and ∼9.8 million comments. We classified the veracity of the posts being discussed as true, mixed, or false using three fact-checking organizations. Regardless of veracity, fact-checked posts had larger and longer lasting conversations than claims that were not fact-checked. Among those that were fact-checked, posts rated as false were discussed less and for shorter periods of time than claims that were rated as true. We also observe that fact-checks of posts rated as false tend to happen more quickly than fact-checks of posts rated as true. Finally, we observe that thread deletion and removal are systematically related to the presence of a fact-check and the veracity of the fact-check, but when deletion and removal are combined the differences are minimal. Theoretical and practical implications of the findings are discussed.

**Significance Statement**

False stories shared on Twitter diffuse more rapidly and widely than true stories. However, whether this pattern holds across other social media platforms is an open question. Here, we investigate user engagement with posts eliciting fact-checking comments on Reddit. In contrast to prior work, we find that posts eliciting comments that include fact-checks indicating the information is true tend to receive more engagement across a variety of metrics than do posts eliciting comments that include fact-checks indicating the information is false. This result is consistent with the interpretation that there are important platform-level differences in how message veracity influences engagement and diffusion.

## Introduction

The flow of misinformation online is a prominent topic in the social and information sciences (1–5). A perennial concern in this domain is the idea that falsehoods travel quickly online (6, 7). Recently, scholars have demonstrated that tweets eliciting a reply that includes a fact-check labeled false by fact-checkers travel farther and faster than tweets eliciting a fact-check labeled true (8).^a^ Furthermore, these scholars argue that this pattern is shaped more by humans’ attention to novel information than by individual characteristics, social norms, network structure, the presence of bad actors, among other possible factors.

These insights are undeniably important, but does this pattern reflect an underlying principle of information flowing across social media? That is, does the virality of fabricated claims transcend the space in which they are shared or the attributes of the users who share them? This would be consistent with the assertion human nature best explains the pattern: give people a way to share messages over long distance, at great speed, and with little cost, and falsehoods will inevitably rise to the top. There is, however, evidence that the relationship between human nature and the capabilities afforded by technology is often more complex (9, 10). In this project, we ask whether the pattern observed on Twitter might be in part shaped by other factors, including technical features of the platform, social norms among user communities, and demographic characteristics of users. To begin to answer this question, we examine user engagement with posts eliciting fact-checking comments on Reddit, another social media platform widely used for news in the USA, but which has characteristics that are quite different from those of Twitter.

The two platforms differ in terms of their technical attributes, their social norms, and their userbase. Twitter is often characterized as a tool for self-broadcasting (11). User posts, or tweets, can elicit a variety of responses, including replies and retweets, but most generate no reaction at all and extended discussions are rare (12, 13). This is likely partly due to the way the platform handles interactions. For example, top-level tweets are always prominently displayed, and responses to them are shown using single-level threading, meaning that there is no built-in mechanism by which users can respond to anything other than the original tweet. In contrast, complex threaded discussion is a defining feature of Reddit. Users share content, both original and from other sources, in communities of shared interest. It is common for other users to respond, often engaging in sustained discussion with the original poster and with one another (14). This style of communication could help shift users’ attention from their social identity, which tends to take precedence on (semi-) anonymous online platforms such as Twitter and Reddit, toward a more individual identity. Other scholarship suggests that such a shift would have implications for how users interact with one another, potentially promoting more civil interactions (15). Reddit also allows users to rate posts and uses these ratings to order the display of posts. Technical attributes such as these often influence communication practices in important ways (16–18).

Social conventions for communicating on the two platforms are also distinct. Twitter is well known for its short-message format; it is not known as a place to find rich, substantive conversations (19). This may be due in part to the fact that incivility on the platform is rampant, disincentivizing more thoughtful interaction (13). Although the company that operates the social media platform has rules prohibiting some types of speech (e.g. you may not threaten violence against an individual or group, https://help.twitter.com/en/rules-and-policies/twitter-rules), there are no guidelines promoting thoughtful or deliberative discussion. More recently, Twitter has begun to police misinformation on its platform, labeling or even removing false content that is considered dangerous (20), but these policies do not apply to the more mundane political falsehoods that regularly circulate on the network.

In contrast, on Reddit, each community, known as a subreddit, prominently features a statement that characterizes its mission and explicitly lays out rules describing the types of interactions allowed and/or prohibited. In many political communities, these guides actively encourage thoughtful dialogue. For example, posts in the subreddit known as r/politics must concern current US politics, and conversations must be civil and constructive. Further, these rules are policed by moderators who have the authority to remove posts that violate them and who will sometimes ban users who consistently fail to respect community expectations. The influence of these rules and norms on Reddit discussion is well documented (21). Furthermore, although there is significant variation across subreddits, there are some macro-level norms that are respected throughout the site (22).

There are also important differences in who uses the two platforms. In 2020, Twitter was used by about 25% of Americans, while Reddit was only used by about 15% (±1.7%) (23). This gap is even larger when focusing on news use: 15% Americans say they regularly consumed news on Twitter, versus only about 6% who report regularly consuming news on Reddit. There are also important demographic differences between users of the two platforms. Reddit users are more likely than Twitter users to be male (67% vs. 54%), they tend to be younger (92% of Reddit users are under the age of 50 vs. 76% of Twitter users), they are slightly more likely to be White (59% vs. 54%), and they tend to be more liberal (79% Democrats vs. 65% Democrats).

All of these differences could have significant implications for user engagement with false claims. On Twitter, where messages are more likely to be broadcast than discussed, interactions are often uncivil, community norms are weak and difficult to police, and the userbase is more diverse, it is easy to imagine how false news stories, which are often more novel and shocking than true ones, could generate unusually high levels of engagement (8). Reddit, in contrast, is composed of communities of interest, many of which work to promote discussion that is both civil and on-topic. Users with a vested interest in constructive conversation and who hope to better understand the issues being discussed as a result would have good reason to attend to the accuracy of the content shared. False news stories might garner more engagement initially, by virtue of their novelty and shockingness, but these same attributes could encourage skeptics to seek verification, and to share what they find. Once fact-checked, Reddit users might be more likely to respond by abandoning claims shown to be false while continuing to discuss those grounded in truth.

In sum, we assert that the propensity to share false news stories farther and more rapidly than true stories on Twitter may be less about fundamental aspects of human nature or of social media than it is about the complex interaction between human decision making, community norms, user characteristics, and attributes of the technologically mediated environment.

To test this claim, we examine engagement with posts eliciting fact-checking comments on Reddit over a three-year period, assessing whether the patterns observed on Twitter replicate on this social networking platform. Following prior research, we use fact-checks posted in these conversation threads to classify the veracity of the content shared (8). We find that claims posts eliciting fact-checking comments that indicate the fact-checked information is false were discussed less and for shorter periods of time than posts eliciting fact-checking comments that indicate the fact-checked information is true. The fact that this pattern differs from the one observed on Twitter is significant, though our data do not allow us to say why this occurs. In the Discussion section, we briefly consider how future research may enable us to better understand the sources of these platform-level differences. Further, we find that the rate of engagement with a post declines rapidly after a fact-checking comment that indicate the fact-checked information is false is introduced to the conversation. Finally, we investigate whether the deletion or removal of posts may contribute to these patterns.

## Materials and methods

**Fig. 1.**
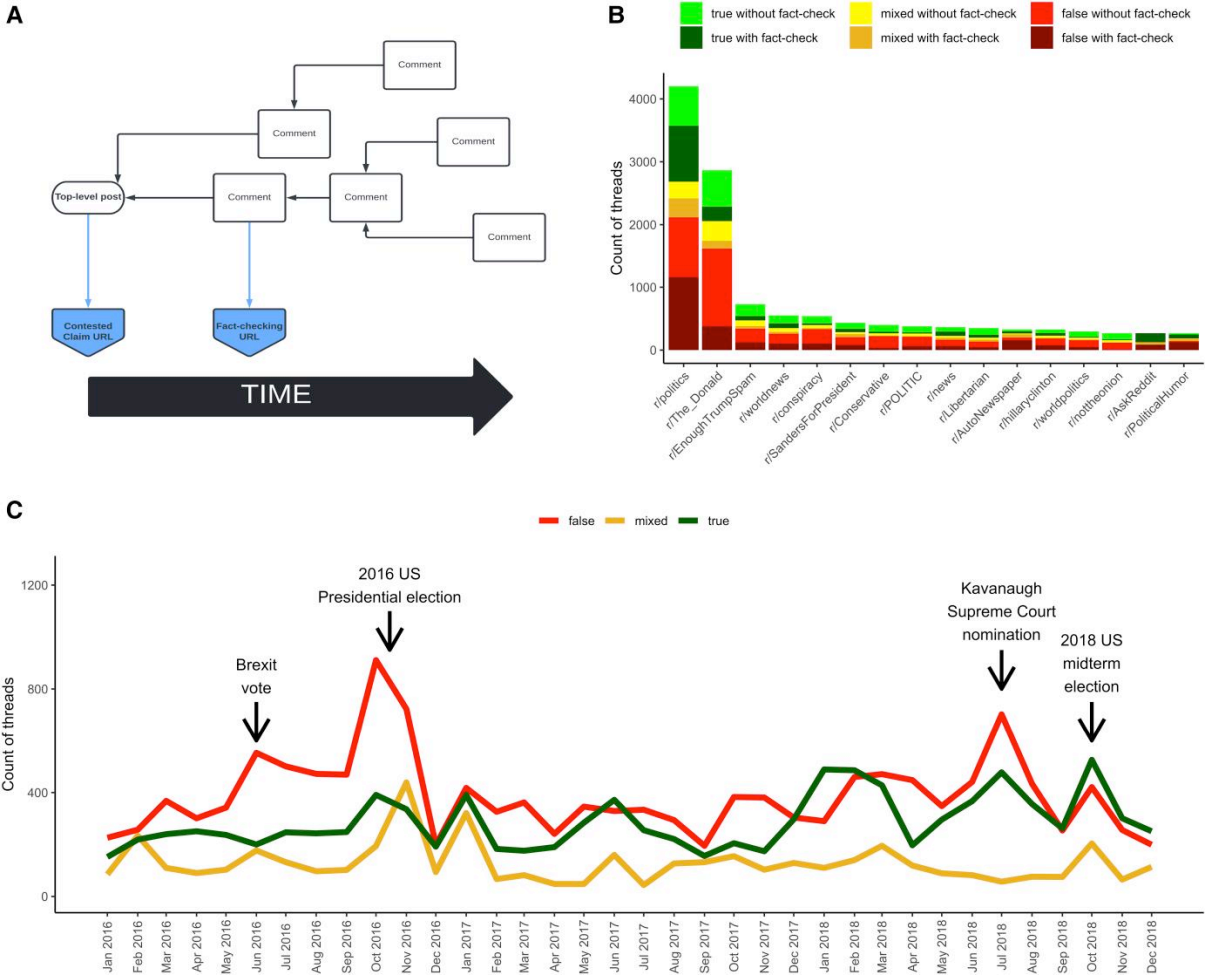
Thread structure, most frequent subreddits, and rates of threads over time. Panel A shows an example of a thread in which the top-level post links to somewhere else on the Internet and a comment replying to the post includes a link to a fact-checking organization. Panel B shows the distribution of threads that had been fact-checked somewhere on Reddit across subreddits, broken out by veracity and whether or not a fact-checking comment is in the thread. Panel C shows the counts of true, mixed, and false threads by month over the course of the study period.

Here, we investigate the differential engagement with and response to posts eliciting fact-checking comments that indicate the fact-checked content is true, posts eliciting fact-checking comments that indicate the fact-checked content is false, and those that elicit fact-checking comments that indicate the fact-checked content is partially true and partially false (“mixed”) on Reddit. To do this, we first collected all posts and comments from Reddit from 2016 through 2018. We then searched the comments that directly replied to a post for links to three fact-checking organizations (PolitiFact, Snopes, and the Washington Post Fact Checker). Because our focus is on political posts, and Snopes classifies claims across many areas, we only included claims which Snopes classified as related to politics. We next ensured that all top-level posts included for further analysis included a link to an external website (i.e. not a link to another page on Reddit), consistent with our goal of assessing the virality of content produced elsewhere and shared on Reddit. Each fact-checking comment labels the associated post as true, false, or mixed. For convenience, and following prior work (8), we refer to the thread by the veracity label applied to the post assessed in the fact check. Although we do not believe that this label captures the accuracy of the top-level post or necessarily its linked URL with perfect fidelity, we argue that it is a reasonable proxy. For example, a fact check might correct a false claim made by the original poster alongside an accurate news story, but such exceptions are unlikely to be systematically biased by veracity. We then collected the veracity rating that the fact-checking organization reported for each of the linked claims. Next, we collected all of the comments in the comment tree that resulted from the posts, including links to news. This enabled us to create a data set of 10,308 posts that included links to news stories that were discussed in 10,785 comment threads that could be classified by veracity. In total, these posts that included links to news stories received ∼8.3 million comments from ∼783,000 unique comment authors. Fig. 1A shows an example of the structure of a thread that elicits a comment with a link to a fact-checking organization.

We next collected all posts that included a link (URL) to the same news story as one of the news stories that had been classified by its veracity regardless of whether the post elicited a comment linking to a fact-checker. This process enabled us to collect an additional 18,073 threads that had the links to news that had been linked to in threads that include a fact-checking comment using the above process.^b^ These posts received ∼1.5 million comments from ∼222,000 unique comment authors. Taken together, the full data including posts that included links to news stories with direct comments that link to fact-checkers and posts that included links to news stories without such comments comprise ∼29,000^a^ posts with ∼9.8 million comments from ∼849,000 unique comment authors. Importantly, the way in which we categorize posts by veracity means that we will only identify news stories that a user has linked to a fact-check in a comment on Reddit. For all types of news, this means that high-profile news that includes ambiguous content is most likely to be checked. That is, true news stories that are very obviously true are unlikely to be included, as are false news stories that are so obviously false that a fact-check is not necessary. Finally, the claims being made in the story must be verifiable in some manner. Stories for which fact-checkers have no way of assessing the veracity of the claims being made would not be checked. The stories included in this study, therefore, occupy a middle ground in which the claims being circulated were plausible, but not obviously true or false, and for which a veracity rating was possible.

One of the primary features of Reddit is the organization of information into subreddits, or subforums. Fig. 1B shows the subreddits that received at least 200 posts that included links to news stories that were classified based on the veracity of the URL in the post, separated by veracity and whether or not the thread elicited a fact-checking comment. The figure shows that of the total of the 2,437 subreddits that received at least one post, two subreddits, r/politics and r/The_Donald, received a disproportionate number (24.5%). Further, subreddits vary significantly in both the rate of posts that included links to news that was rated as true or false being posted as well as whether or not the threads receive fact-checking comments. For example, 61.6% of the r/conspiracy threads were to posts that included links to news that in which the fact-checking comment suggested the information was false of which 30.7% were fact-checked, while 32.0% of the r/AskReddit threads were to posts that included links to news that in which the fact-checking comment suggested the information was false and all of these were fact-checked. Table S1 in the Supplementary Appendix provides information on the veracity and rate of fact-checking comments in all of the top 25 subreddits.

To quantify engagement with each of the posts, we defined four measures of engagement. First, we counted the number of comments that a post received as a measure of activity. Second, we counted the number of unique commenters who had made at least one comment on the post as a measure of the size of the conversation. Third, we measured the maximum depth of the comment tree (the maximum number of steps between a comment and the original post—e.g. a comment on a comment on a comment on a post would be at depth three) as a measure of the extent to which the conversation went back and forth between conversation participants. Last, we measured the lifetime of the post in hours as the difference between the time the first and last comments are made as a measure of how long-lasting the conversation is.

Finally, Reddit enables the elimination of content through two processes. First, users may delete their own prior posts or comments. Second, moderators may remove content, typically because it violates a rule or norm in the given community. When content is deleted or removed, the content of the post is no longer available (it is replaced with either “[deleted]” or “[removed]”), but a placeholder for the content is still available. As such, we collected information on whether posts that included links had received a fact-checking comment somewhere on the site were deleted or removed to try to understand whether these processes may contribute to patterns of engagement with posts that include such links.

## Results

The degree to which posts that were fact-checked are shared and discussed on the site varies considerably over time. Fig. 1C shows the number of true, false, and mixed posts that were fact-checked on Reddit in each month of the study period. As the figure shows, there is substantial variability in the number of threads that include a link to a news story that was fact-checked, and the sharing of posts that were fact-checked is highly responsive to high-profile political events. Although both posts that were fact-checked in which the fact-checked information was rated as true and rated as false tend to be posted more frequently surrounding high-profile events, posts that were fact-checked and the fact-checked information is false tend to be more frequent in the periods surrounding these events, with a high peak surrounding the 2016 US presidential election in particular. As the figure shows, throughout most of the period of observation (and overall) the number of posts that elicit a comment including a fact-checking link we identify as false is higher than the number of posts that elicit a comment including a fact-checking link we identify as true. However, as discussed in detail below, the rate of engagement with posts eliciting a comment including a fact-checking link identified as true is higher.

As a post that includes a link to a news story receives comments, the activity, size of the conversation, the depth of the comment tree, and the lifetime of the conversation increase. However, there are substantial differences between posts in which a fact-check occurs and those in which one does not. Posts with fact-checks receive substantially higher rates of engagement than those without fact-checks. Furthermore, differences in engagement with posts that were fact-checked and the fact-checked information is false versus true are more pronounced when a fact-check is present.

**Fig. 2.**
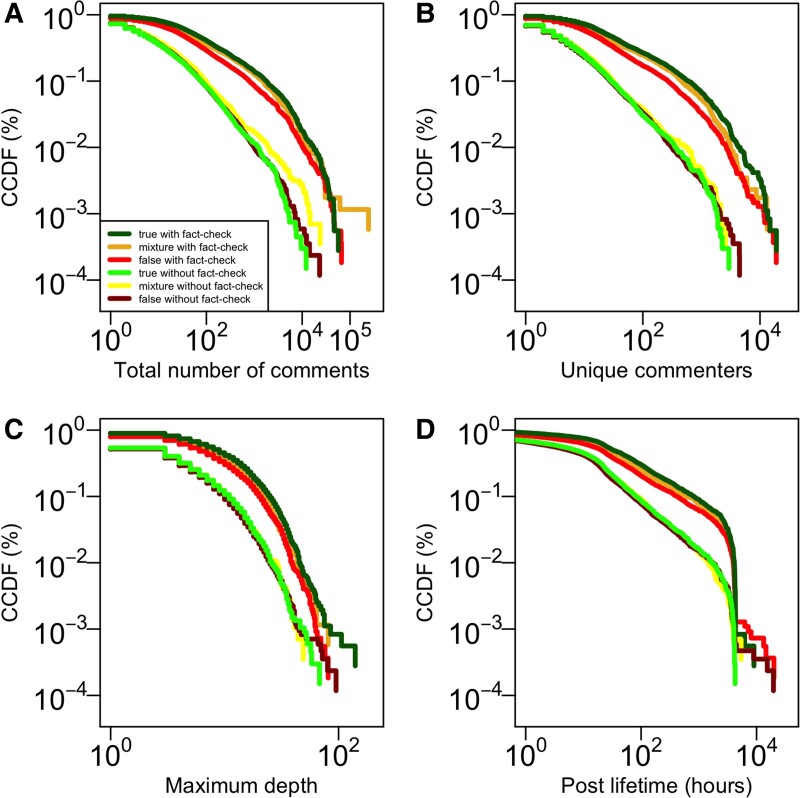
Distributions of comments, commenters, thread depth, and post lifetime by thread veracity. Complementary cumulative distribution functions (CCDFs) of conversations of news stories that did receive a fact-checking comment (A) Size, (B) Activity, (C) Maximum depth, and (D) Lifetime.

We found that across all four measures of engagement with posts that were fact-checked, those in which the fact-checked information is true received more comments than those in which the fact-checked information is false [Kolmogorov–Smirnov (K-S) tests are reported in Tables S2 to S9]. On average, posts in which the fact-checked information is true received 929.65 comments while posts in which the fact-checked information is false received 581.76 comments (Fig. 2A). Similarly, posts in which the fact-checked information is true received comments from an average of 285.84 unique commenters while posts in which the fact-checked information is false received comments from 152.73 unique commenters (Fig. 2B). A similar relationship is observed for the maximum depth of the comments, as posts in which the fact-checked information is true went to an average maximum depth of 11.62 while posts in which the fact-checked information is false went to an average maximum depth of 8.94 (Fig. 2C). Finally, posts in which the fact-checked information is true had conversations that lasted longer with an average lifetime of 323.19 h while posts in which the fact-checked information is false had an average lifetime of 234.57 h (Fig. 2D). In all cases, these differences were statistically significant at the *P* < 0.01 level. See the Supplementary Appendix for more details.

One of the defining features of Reddit that may explain some of the differences in engagement that posts in which the fact-checked information is true or false receive on the site is the voting feature. On Reddit, posts and comments receive up and down votes that are then aggregated into a composite score that is up-votes minus down-votes. The score of a post or comment can affect whether a post is likely to be shown at all or whether it is shown at the top of a ranked set of content on the site. We first investigated the patterns of the scores of posts that had been fact-checked. We found that posts that had a fact-checking comment that indicated the fact-checked information was true had significantly higher scores on average (*M* = 3509.94) than posts that had a fact-checking comment that indicated the fact-checked information was false (*M* = 1769.72, *P* < 0.01). See Table S14 in the Supplementary Appendix for details on the scores that posts received. It is important to note that we are not able to observe the timing of votes, and as such we are not certain whether posts in which the fact-check indicates the information is true receive more engagement or if highly engaged with posts that score highly are more likely to receive fact-checks, perhaps with true content being especially so.

We next investigated the scores of fact-checking comments. We found that comments that linked to a fact-checking source that indicated that the fact-checked information was true received significantly higher scores on average (*M* = 41.54) than comments that linked to a fact-checking source that indicated the fact-checked information was false (*M* = 26.57, *P* < 0.01). See Table S15 in the Supplementary Appendix for details on the scores that fact-checking comments received. Similar to the above, we are uncertain about the causal direction. It may be the case that posts that contain true information receive many views and thus the comments on them are more likely to be viewed and voted on, perhaps positively, than are posts that contain false information. If so, it may be the case that the pattern of votes on fact-checking comments is in part due to the rates of engagement with posts that contain true or false information, as described above. Another possibility is that fact-checking comments, and in particular the veracity that the comment indicates, has a strong effect on subsequent engagement. Unfortunately, Reddit does not make the timing of votes available in an easily accessible way, so understanding the causal effect of fact-checking comments on scores would require another research design. However, the timing of comments on posts is readily accessible, and as described below, these data enable us to have some understanding of how fact-checking comments may affect engagement on the site.

The relationship between the veracity of the fact-checked post and the degree of engagement is much less strong in threads that do not have a fact-checking comment. Among threads that did not receive a fact-check, we found little substantive difference in the rate of engagement [Kolmogorov-Smirnov (K-S) tests are reported in Tables S10 to S13]. On average, posts in which the fact-checked information is true received 61.31 comments, whereas posts in which the fact-checked information is false received 86.83 (Fig. 2A), and the difference was not statistically significant (*P* = 0.43). Similarly, posts in which the fact-checked information is true received comments from an average of 21.91 unique commenters, whereas posts in which the fact-checked information is false received comments from 22.05 (Fig. 2B), and the difference was not statistically significant (*P* = 0.07). A similar pattern was evident for the maximum depth of the comments, as posts in which the fact-checked information is true had an average maximum depth of 4.48, whereas posts in which the fact-checked information is false had an average maximum depth of 4.22 (Fig. 2C), though in this case the difference was statistically significant (*P* = 0.01) the size of the difference is much smaller than in threads that elicited a fact-checking comment. Finally, posts in which the fact-checked information or false had conversations that lasted longer with an average lifetime of 70.10 h while posts in which the fact-checked information is true had an average lifetime of 69.57 h (Fig. 2D), and again although the difference was statistically significant (*P* < 0.01) the average difference is of a relatively small magnitude. See the Supplementary Appendix for more details.

**Fig. 3.**
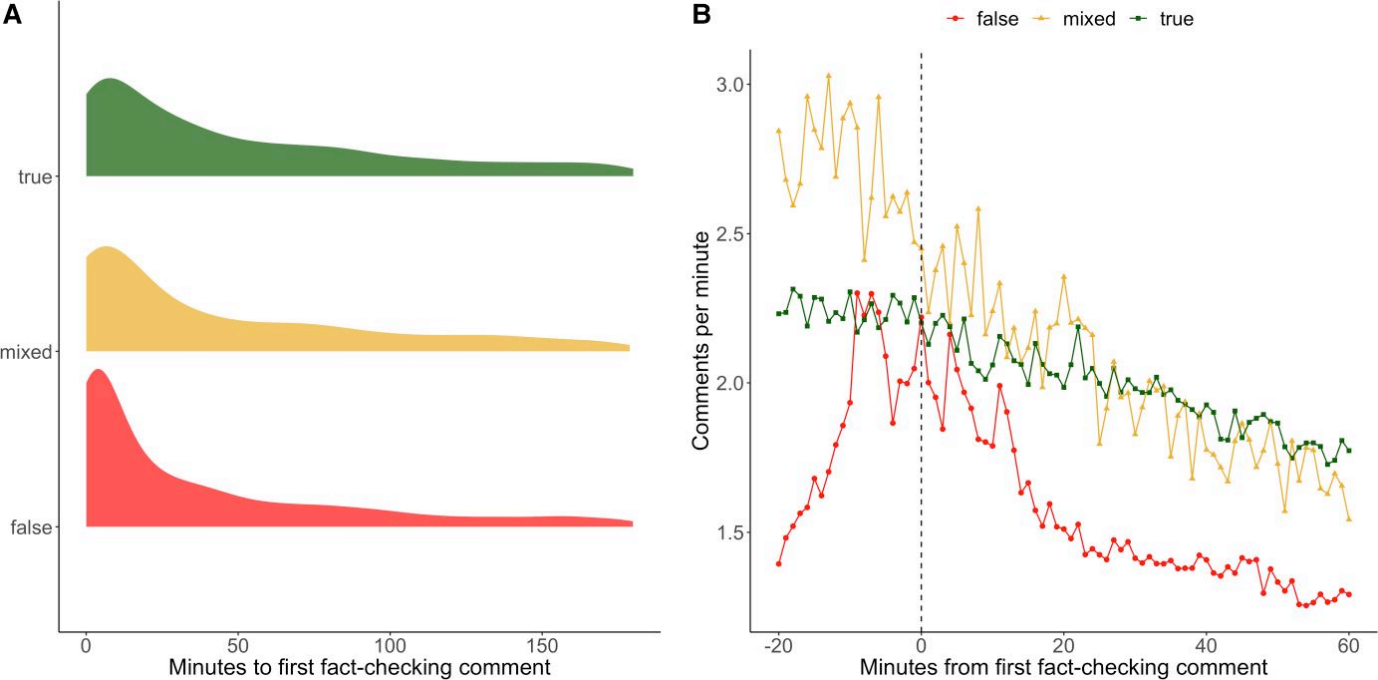
Distribution of time to and rate of commenting prior to and after first fact-checking comment. Panel A shows the distribution of the number of minutes until the first fact-checking comment on a post was made for the three veracity ratings of posts. Panel B shows the average number of comments per minute for each minute relative to the fact-check (e.g. −20 is 20 min before the fact-checking comment is made, and 20 is 20 min after the fact-checking comment is made). We note that Panel A includes only those threads that received a fact-checking comment within 6 h of the initial post for the purposes of illustration (more than 75% of posts met this criteria).

We next investigated the rate of activity on posts temporally adjacent to the posting of a fact-check. These analyses are limited to the 10,785 threads in which a fact-checking comment directly replied to a post. Fig. 3A shows the distribution of time between a post being created and the first comment that includes a fact-checking link. It typically took users about half as long to share a fact-check in in which the fact-checked information is false as to one in which the fact-checked information is true. The median time to the first fact-check of a post in which the fact-checked information is false was 63.84 min, while the median time to the first fact-check in which the fact-checked information is true was 116.73 min. A Kolmogorov–Smirnov test of the difference in the distributions of first fact-checking comment times showed that this difference was statistically significant (*D* = 0.13, *p* < 0.01). Fig. 3B shows the rate of comments on posts in the minutes leading up to and away from the first fact-check. While engagement with posts in which the fact-checked information is true or mixed was substantively unchanged by the inclusion of a fact-check, results suggest that a fact-checking comment marks an important inflection in the commenting rate. Prior to a fact-check being posted, discussion of posts in which the fact-checked information is false accelerated rapidly until it was comparable to the rate associated with posts in which the fact-checked information is true. After the fact-checking comment was posted, however, the commenting rate about posts in which the fact-checked information is false dropped off more quickly than either posts in which the fact-checked information is true or mixed.

It is important to note that the causal direction of the relationships observed in Fig. 3B is difficult to know without further investigation. It is possible that increased engagement drives people to perform fact-checks and therefore content that users view as likely to garner further attention if not challenged may be more likely to receive a fact-checking comment. It is also possible that fact-checking comments reduce engagement after the fact, as suggested by the figure. A third possibility is that fact-checking comments may be more likely to be made in some communities, perhaps especially in communities where the fact-check is more likely to be well received. In short, although the data we have collected is suggestive that fact-checking comments may slow engagement with false content, further investigation is needed to better understand these relationships.

**Fig. 4.**
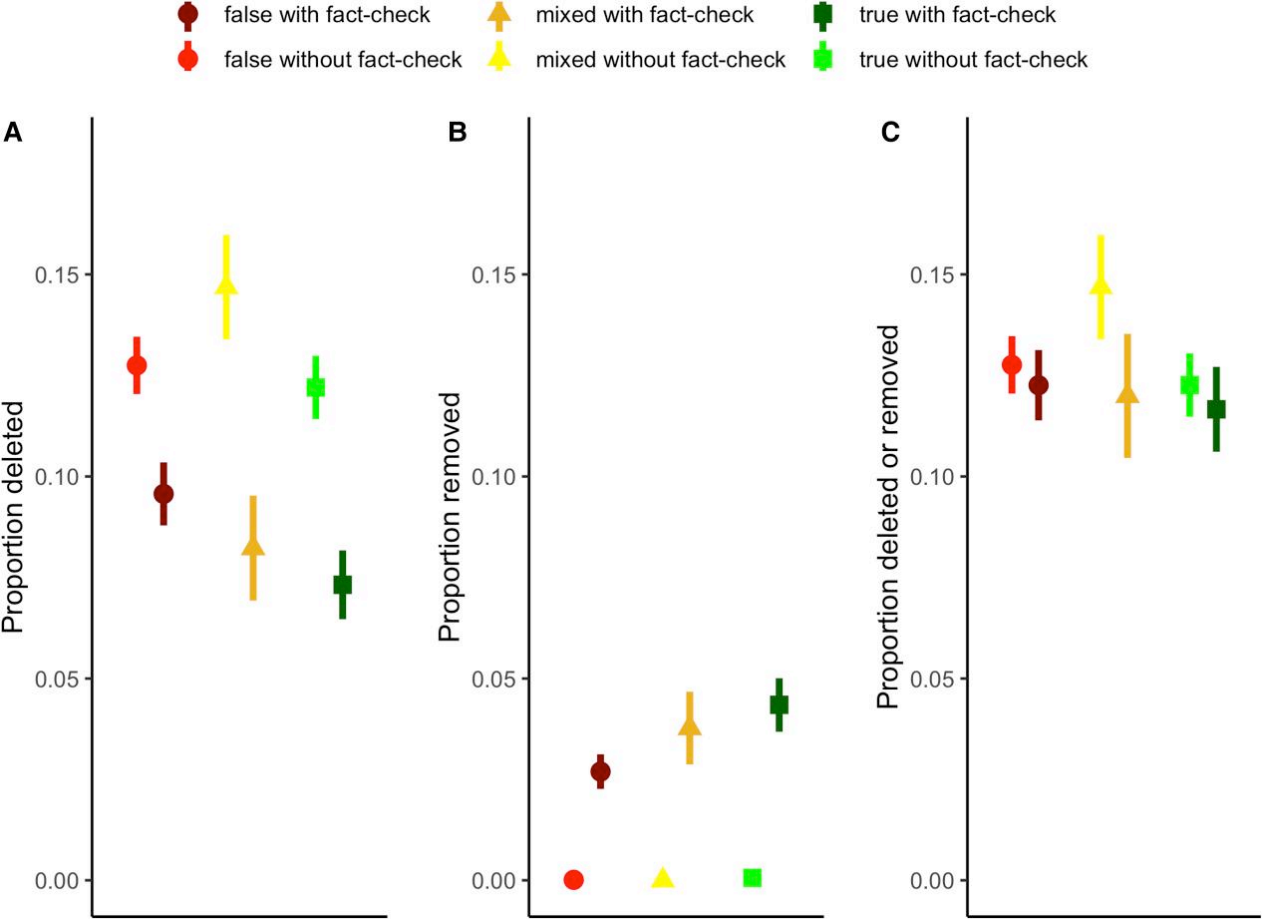
Frequency of post deletion and removal. Panel A shows the proportion of posts that were deleted by the author separated by veracity rating and the presence or the absence of a fact-checking comment. Panel B shows the proportion of posts that were removed by the someone other than the author, typically a moderator, separated by veracity rating and the presence or the absence of a fact-checking comment. Panel C shows the proportion of posts that were deleted by the author or removed by someone other than the author separated by veracity rating and the presence or the absence of a fact-checking comment.

One factor that may contribute to differences in the level of engagement with posts concerns the deletion or removal of the original text that accompanied a post. On Reddit, a post may include a title, a URL that the title links to, and an optional message written by the user creating the post. After the post has been created the same user may delete the post or a moderator may remove the post. When this occurs, the text provided by the user no longer appears and is replaced by either “[deleted]” (if the user self-deletes their own post) or “[removed]” (if a moderator removes the post of another user). Fig. 4A shows the rate by which posts were deleted by the posting author by the veracity of the link in the fact-checking comment as well as whether the post received a fact-checking comment. Posts receiving a fact-checking comment were significantly *less* likely to be deleted by the original user overall (*t* = 11.57, *P* < 0.01). Interestingly, the pattern was the same regardless of veracity: false (*t* = 5.91, *P* < 0.01), mixed (*t* = 6.91, *P* < 0.01), and true (*t* = 8.27, *P* < 0.01) veracity ratings. Fig. 4B shows the rate by which posts were removed by a moderator by the veracity of the link in the fact-checking comment as well as whether the post received a fact-checking comment. Posts receiving a fact-checking comment were significantly *more* likely to be removed by a moderator overall (*t* = 19.31, *P* < 0.01). This, too, was unaffected by veracity: false (*t* = 12.22, *P* < 0.01), mixed (*t* = 8.22, *P* < 0.01), and true (*t* = 12.55, *P* < 0.01). However, it appears as though the removal and deletion processes may balance each other out. When the two measures are combined into a single measure of whether a post has either been deleted or removed, the rates are comparable across both veracity and the presence or absence of a fact-checking comment. Fig. 4C shows the proportion of posts that were removed by a moderator or deleted by the original author by the veracity of the link in the fact-checking comment as well as whether the post received a fact-checking comment. In these comparisons, there is no significant difference between posts identified as true or false via fact-checking comments depending on the presence of a fact-checking comment, though for mixed stories the rate or deletion or removal is modestly higher among posts without a fact-checking comment.

## Discussion

Analyzing three years of discussion on Reddit, we find that fact-checked posts in which the fact-checked information is false have a prominent role on the platform, especially in well-known political subreddits, such as r/politics and r/The_Donald. Further, we find that discussion threads that include a link to a fact-checking site tend to include more comments, from more unique commenters and over a longer period of time than threads that do not. Most importantly, and in contrast to analysis of data collected on Twitter, we find that fact-checked posts in which the fact-checked information is false have much lower engagement than fact-checked posts in which the fact-checked information is true. There are a number of possible explanations for this. Perhaps most obviously, it may be due in part to the attenuating effect that being labeled “false” by a fact-checker has on users’ rate of commenting on messages. A similar pattern has been observed on Facebook (24).

Evidence that false news stories capture more attention on Twitter than true ones is undeniably troubling (8). The platform is a regular source of news for millions of Americans (23) and it serves as a bully pulpit for many well-known American politicians (25). There is, however, risk in painting with too broad a brush. Behaviors observed on Twitter are not necessarily representative of behaviors on other social media platforms or on the Internet more generally. Analyses reported here show that the two platforms are sometimes used in strikingly different ways. Our data do not allow us to say why these differences exist, but we have identified a wide range of potential contributors, including differences in the platforms’ technical architectures, social structures, and userbases. More research is required to understand how each of these attributes shape the role that veracity plays in the flow of information.

As noted above, we classify all posts that include a link to a website outside of Reddit by veracity based on direct comments to top-level posts that include a link to a fact-checking website. Although our focus here has been on the veracity of the posts that include news that is in the linked content, it is also possible that the comment linking to a fact-checker is providing a fact check on user-generated content in the top-level post, is replying to another comment even though it is a direct comment on the top-level post, or is unrelated to prior content. Future work may wish to further investigate fact checking of various kinds of content on social media.

We briefly consider a few possible avenues for future research. Although the relationships observed are important in themselves, establishing the direction of causality would be valuable. The primary goal of this work was to understand the extent to which discussions about posts that include true and false claims vary, but we also found evidence suggesting that fact-checks are not merely indicators of veracity, but may also alter subsequent conversation. It would be important to understand whether, for example, comments offering evidence that a post or news story is false causes the rate of engagement with a post to slow on Reddit and elsewhere. Relatedly, comments that include a fact-check might alter the nature of subsequent comments. For example, perhaps such comments elicit meta-discussion, focused on the fact-check itself. A fact-checking comment might also prompt more substantive discussion, spurred on by the new information provided. Or, alternatively, perhaps fact-checking comments disrupt the discussion, resulting in conversations that are less wide ranging. In sum, there is much left to understand about how individuals engage with news and fact-checks online.

It would also be worth examining the relationship between user demographics and veracity-based differences in engagement. Compared to Twitter, Reddit’s userbase is younger and more likely to be male (27, 26). Such difference could influence which posts are fact-checked and how users respond to those fact-checks. There are also important differences among the various communities that exist on Reddit. The social media platform explicitly encourages users to create and to seek out communities (subreddits) in which they may find and interact with other users who share their interests, viewpoints, etc. This suggests that there are likely to be meaningful differences across these communities in terms of their demographics, the standards and social conventions to which users are expected to conform, and the extent to which these norms are policed. Research examining inter-community differences in news diffusion would be valuable.

We also observe that the rate at which dubious political news is posted to Reddit differs significantly over time, and tends to increase surrounding high-profile political events. This is consistent with prior work on the site that showed that there were higher rates of cross-ideological interaction surrounding high-profile events (14). In the case of the posting of dubious news, these results highlight the need for platforms and users to be vigilant when high-profile events take place, as the news posted to the site is likely to include substantial amounts of both true and false information.

The role of the removal and deletion of content in affecting the engagement other users have with posts eliciting comments including fact-checking links indicating the information in the post is true or false is as-yet uncertain. Although we found that posts without a fact-checking comment were more likely to be deleted and posts with a fact-checking comment were more likely to be removed, in the aggregate the deletion and removal of posts seem to even out. This suggests that it is possible that fact-checking comments draw the attention of moderators to content that would have otherwise eventually been deleted by the author. However, more research is needed on these processes, particularly to understand whether the deletion or removal of content stymies subsequent engagement in meaningful ways that inhibits the spread of false stories.

## Conclusion

This research suggests that engagement with contested posts that include links to news stories circulating on social media is not constant, and that broader diffusion of inaccurate claims is not a universal characteristics of social media; instead, there are important differences across social media platforms. As we further develop our understanding of how such true and false information either takes hold or fails to do so we must understand how factors such as technical affordances, social norms, and differences in user populations separately or together contribute to false information capturing the public’s attention.

## Supplementary Material

pgad018_Supplementary_Data

## Data Availability

The data underlying this article are made available in the Harvard Dataverse, https://doi.org/10.7910/DVN/E4FHIZ.

## Notes

a The authors of that work refer to the news stories linked in the tweets they analyze as “false news” and “true news”, respectively. We take a somewhat more conservative approach and describe the behavior of commenters as it relates to the inclusion of links to fact-checking organizations rather than attributing the veracity label to the URL included in the original post.

b We note that we did not verify that the fact-checks covered the same topics as were covered by the news stories in the original URL. Prior work (8) used a text similarity measure to verify that fact-checks of content on Twitter were about the original post’s content at scale. However, on Reddit messages tend to be longer and more complex than on Twitter, which makes this approach less tenable. It is possible that the fact-checks are about content in the parent post, and not the linked news article. In these cases, our approach to classifying URLs is conservative, and would work against our ability to detect differences between posts that include URLs classified as true and false.

c In total, we identified 13,961 threads that were classified as false, 10,297 that were classified as true, and 4,600 that were classified as mixed.
