# Supplementary material for: Engagement with fact-checked posts on Reddit
Source: PNAS Nexus. 2023 Jan 27;2(3):pgad018. doi: 10.1093/pnasnexus/pgad018 (PMC10011804; doi:10.1093/pnasnexus/pgad018)
Supplement: pgad018_Supplementary_Data [file pgad018_supplementary_data.pdf]

Supplementary information for: Engagement with fact-checked posts on Reddit

Robert M. Bond[1*] and R. Kelly Garrett[1]

[1]School of Communication, The Ohio State University, Columbus OH 43210, USA.

[*]To whom correspondence should be addressed. E-mail: bond.136@osu.edu

Data

News stories are frequently shared on Reddit by a user to begin a thread of conversation about the news. These stories are shared when a user creates a top-level 'post' on the site. The post takes the form of a link to a page on which comments on the post may be made by any user. A post may be a link to another place on the internet or it may be simply text that the initial user provides. As users comment on a post, they may do so in a tree-like fashion in which initial comments are to the post itself, but subsequent comments may reply to the initial post or to comments on the post, to comments on comments, and so on.

In some instances, users may comment on an initial post with a link to a fact checking organization, presumably to add a third-party's veracity rating to the information. We searched for posts in which a top-level comment (a comment replying directly to the post) contained a link to a fact checking organization to identify contested news stories that were being shared on Reddit.

Data collection on contested news stories

To identify contested news stories, we rely on links to fact checking organizations. As such, we selected three fact checking organizations that are known to thoroughly check contested news stories that are circulating – Snopes, Politifact, and the Washington Post Factchecker. We collected all articles from

these fact-checkers, and parsed the resulting HTML files for the fact check rating associated with the contested news story being evaluated. We normalized these ratings to be on a five-point scale ("False", "Mostly false", "Mixed", "Mostly true", and "True"). Following previous work (1), for analysis we collapse these into a three-point scale in which "False" and "Mostly false" contested news stories are treated as "False" and "Mostly true" and "True" contested news stories are treated as "True". Mixed contested news stories are relatively rare, both for fact checkers and among those that are shared online. If any story received multiple fact checks that disagreed, that story was removed from the data.

The Washington Post Factchecker and Politifact focus nearly exclusively on contested news stories that relate to politics. Snopes, however, checks contested news stories across a wide array of subject areas. Because our work focuses on political contested news stories, we used Snopes' own categorizations of the contested news stories they had checked to identify political contested news stories. We only use Snopes fact checks that are categorized as relating to "Politics" and "Politicians", as these should certainly be contested news stories that relate to politics.

Data collection on Reddit

We searched a nearly complete database of all posts and comments made to Reddit to that covers January 1, 2016 through December 31, 2018 to identify comments containing links to fact checking organizations. We identified all posts in which a top-level comment contained such a link. We then collected the top-level post itself as well as all comments to that post.

Similar to an approach used in prior work (1), we took care to make sure that the comments containing links to the fact checkers were addressing the claims made in the top-level post. First, we only included posts that contained a comment with a fact checker that was a directly reply to the top-level post itself. There are many instances in which commenters deeper into the comment tree link to fact checkers. However, it is far less clear whether these commenters are responding to the information

contained in the top-level post or to information contained in a subsequent comment. We also removed instances in which the top-level post itself contained a link to a fact checking organization. Although we believe that research into how fact checking organizations themselves are discussed on the site is needed, that question is beyond the scope of this research. We next removed any posts in which the top-level post that contained a link to a fact checking website contained a link to a non-fact check or to a fact check with a rating that did not contain a veracity rating (e.g., a rating of "inconclusive" or "not enough information").

We next endeavored to ensure that the information in the top-level post was not in dispute via conflicting fact-checks. We collected the URL that was contained in the post itself and searched across all posts with a fact check rating for any that were to the same URL. In any case in which a URL was repeated, we checked for disagreements in the fact check ratings and removed all instances in which a disagreement occurred (1.3% of URLs contained disagreeing comments). In total, we identified 10,786 top-level posts that had been fact checked directly and were not in dispute.

We next took all URLs from the posts with fact check ratings and searched all other posts on Reddit for top-level posts that contained one of the fact checked URLs. We identified 18,472 such posts. We then collected all comments associated with these posts.

In total, we identified 29,258 posts that were associated with 10,309 unique URLs. There were 14,361 posts that were false, 4,600 that were mixed, and 10,297 that were true that corresponded to 5,154 false, 1,635 mixed, and 3,520 true URLs.

	subreddit	Number of conversations	Percent with fact check	Percent true overall	Percent mixed overall	Percent false overall	Percent true with fact check	Percent mixed with fact check	Percent false with fact check	Percent true without fact check	Percent mixed without fact check	Percent false without fact check
1	politics	4203	55.9	36.2	13.5	50.3	21.1	7.2	27.6	15.0	6.3	22.7
2	The_Donald	2867	25.7	28.3	15.4	56.3	8.0	4.5	13.2	20.3	11.0	43.1
3	EnoughTrumpSpam	731	31.5	35.3	18.1	46.6	9.2	5.7	16.6	26.1	12.3	30.1
4	worldnews	552	37.1	36.6	16.3	47.1	13.4	5.4	18.3	23.2	10.9	28.8
5	conspiracy	541	26.6	26.2	12.2	61.6	4.6	3.1	18.9	21.6	9.1	42.7
6	SandersForPresident	435	42.1	34.5	18.4	47.1	12.0	11.3	18.9	22.5	7.1	28.3
7	Conservative	401	16.7	33.2	11.7	55.1	5.7	2.5	8.5	27.4	9.2	46.6
8	POLITIC	380	23.4	30.5	13.7	55.8	4.2	2.9	16.3	26.3	10.8	39.5
9	news	366	43.4	38.3	16.9	44.8	18.0	8.5	16.9	20.2	8.5	27.9
10	Libertarian	351	33.3	43.6	17.9	38.5	12.5	8.5	12.3	31.1	9.4	26.2
11	AutoNewspaper	330	72.7	19.4	20.3	60.3	9.4	15.8	47.6	10.0	4.5	12.7
12	hillaryclinton	328	39.3	30.2	13.7	56.1	12.8	3.4	23.2	17.4	10.4	32.9
13	worldpolitics	297	24.6	33.0	14.1	52.9	4.4	4.0	16.2	28.6	10.1	36.7
14	nottheonion	270	8.5	41.1	14.8	44.1	4.1	1.1	3.3	37.0	13.7	40.7
15	AskReddit	269	100.0	53.2	14.9	32.0	53.2	14.9	32.0	0.0	0.0	0.0
16	PoliticalHumor	269	81.8	31.6	15.2	53.2	23.8	11.9	46.1	7.8	3.3	7.1
17	esist	246	11.4	41.1	18.7	40.2	3.3	1.6	6.5	37.8	17.1	33.7
18	forwardsfromgrandma	229	95.2	16.2	15.7	68.1	15.3	14.8	65.1	0.9	0.9	3.1
19	WayOfTheBern	225	16.9	43.1	13.8	43.1	7.6	1.8	7.6	35.6	12.0	35.6
20	democrats	220	14.5	39.1	14.1	46.8	4.1	0.5	10.0	35.0	13.6	36.8
21	ChapoTrapHouse	193	21.8	44.0	18.1	37.8	7.3	5.2	9.3	36.8	13.0	28.5
22	PoliticsAll	192	4.7	31.8	13.0	55.2	0.5	0.0	4.2	31.2	13.0	51.0
23	changemyview	188	100.0	52.7	15.4	31.9	52.7	15.4	31.9	0.0	0.0	0.0
24	HillaryForPrison	177	20.3	15.3	14.7	70.1	2.3	5.1	13.0	13.0	9.6	57.1
25	PoliticalDiscussion	172	98.3	45.9	16.9	37.2	44.2	16.9	37.2	1.7	0.0	0.0

Table S1: Top 25 subreddits in number of news stories that were included in the study.

Comparing engagement with posts eliciting fact-checking comments

Measures of conversation characteristics

We compare across conversations using the following measures:

- Size: The size of a conversation is measured by the number of unique users contributing a message in the thread.
- Activity: The activity in a conversation is measured by the total number of unique comments made in a thread.
- Depth: The depth of a comment is the number of edges between the original post and a comment. For instance, a comment on a comment on a comment of a post would have a depth of three. We measure the maximum depth of comments associated with each post.
- Lifetime: The lifetime of a post is the difference in time between when a the first comment is made and when the last comment to that post is made in hours. We note that it is possible that threads will continue to receive comments after our data collection is complete, and so the lifetime of a post may extend beyond the time period of observation.

We calculate each of the above measures for each of the conversations in our dataset. Below, we show the summary statistics for each of these measures across veracity for the size of the conversation in Table S2, for the activity of the conversation in Table S3, for the maximum depth of a thread in Table S4, and for the lifetime of the conversation in Table S5. For each measure, we conducted a two-sample Kolmogorov-Smirnov Tests between false and true conversations. As these tables show, across all four measures true conversations receive, on average, greater engagment than do false conversations.

We found that across all four measures of engagement with the posts eliciting fact-checking comments, true posts eliciting fact-checking comments received higher rates of engagement than did false

	N	Mean	SD	Min	Max
False	13,961	73.21	472.34	1	19,127
Mixed	4,600	96.39	507.76	1	14,292
True	10,297	114.01	640.64	1	19,907

Table S2: Summary statistics for the size of conversations in all conversations.
KS-test for false and true conversations: $D = 0.040, p < 0.01$.

	N	Mean	SD	Min	Max
False	13,961	280.51	2,500.81	1	183,762
Mixed	4,600	471.36	5,990.03	1	291,512
True	10,297	364.28	2,361.20	1	114,998

Table S3: Summary statistics for the activity of conversations in all conversations.
KS-test for false and true conversations: $D = 0.034, p < 0.01$.

	N	Mean	SD	Min	Max
False	13961	6.07	7.51	1	196
Mixed	4600	6.55	7.87	1	100
True	10297	6.97	8.49	1	147

Table S4: Summary statistics for the maximum depth of conversations in all conversations.
KS-test for false and true conversations: $D = 0.048, p < 0.01$.

	N	Mean	SD	Min	Max
False	13,961	134.49	644.89	0.00	22,185.88
Mixed	4,600	142.07	531.71	0.00	9,344.33
True	10,297	158.07	587.57	0.00	18,273.06

Table S5: Summary statistics for the lifetime of conversations in all conversations.
KS-test for false and true conversations: $D = 0.067, p < 0.01$.

posts eliciting fact-checking comments [Kolmogorov-Smirnov (K-S) tests are reported in tables S2 to S5]. On average, true posts eliciting fact-checking comments received a 364.28 comments while false posts eliciting fact-checking comments received 280.51. Similarly, true posts eliciting fact-checking comments received comments from an average of 114.01 unique commenters while false posts eliciting fact-checking comments received comments from 73.21.The same was relationship held for the maximum depth of the comments, as true posts eliciting fact-checking comments went to an average maximum depth of 6.97 while false posts eliciting fact-checking comments went to an average maximum depth of 6.07.Finally, true posts eliciting fact-checking comments had conversations that lasted

longer with an average lifetime of 158.07 hours while false posts eliciting fact-checking comments had an average lifetime of 134.49 hours.

The above tables show the summary statistics across our core measures of attention to conversations across all conversations. However, it is possible that some of the differences that we observe are due to the presence of the fact check in the conversation. In the below tables, we separately report the summary statistics for conversations that contain a fact check and those that do not. Table S6 shows summary statistics for the size of conversations, Table S7 shows summary statistics for the activity in conversations, Table S8 shows summary statistics for the maximum depth of conversations, and Table S9 shows summary statistics for the size of conversations that contain a fact checking comment.

Table S10 shows summary statistics for the size of conversations, Table S11 shows summary statistics for the activity in conversations, Table S12 shows summary statistics for the maximum depth of conversations, and Table S13 shows summary statistics for the size of conversations that do not contain a fact checking comment.

Table S14 shows summary statistics for the scores that posts that received a fact-checking comment received. Table S15 shows the summary statistics that comments that linked to a fact check received. We note here for readers a few features of the scores of posts that may not be obvious. First, at the time of data collection the Reddit API made available the aggregate score for a post (up-votes minus down-votes), but not the raw number of up- and down-votes. As such, controversial posts that receive many up and down votes that mostly cancel one another out may appear similarly on score as do posts that receive much less engagement. That is, a score near zero may indicate many up and down votes or may indicate little engagement at all. Second, as discussed in the main text, the scores for posts are not time-dynamic. It would be possible to call the API to collect the score of a post multiple times and in doing so to observe *changes* in the score that a post or comment receives. However, collecting this kind of time-dymanic data on a large number of posts would be time and resource intensive. Because

of this, we are uncertain about how the score of a post responds to a fact-checking comment or how the score that a post has thus far received could impact the likelihood that a fact-checking comment is made in the first place.

References

[1] S. Vosoughi, D. Roy, and S. Aral, "Report the spread of true and false news online," *Science*, vol. 359, no. 6380, pp. 1146–1151, 2018.

	N	Mean	SD	Min	Max
False	5,466	152.73	727.00	1	19,127
Mixed	1,726	214.33	795.04	1	14,292
True	3,593	285.84	1,050.37	1	19,907

Table S6: Summary statistics for the size of conversations in conversations that had a fact checking comment.
KS-test for false and true conversations: $D = 0.145, p < 0.01$.

	N	Mean	SD	Min	Max
False	5,466	581.54	3049.05	1	72,708
Mixed	1,726	1042.84	9567.55	1	291,512
True	3,593	929.60	3900.27	1	114,998

Table S7: Summary statistics for the activity of conversations in conversations that had a fact checking comment.
KS-test for false and true conversations: $D = 0.134, p < 0.01$.

	N	Mean	SD	Min	Max
False	5,466	8.94	8.92	1	106
Mixed	1,726	10.06	9.64	1	100
True	3,593	11.62	10.58	1	143

Table S8: Summary statistics for the maximum depth of conversations in conversations that had a fact checking comments.
KS-test for false and true conversations: $D = 0.134, p < 0.01$.

	N	Mean	SD	Min	Max
False	5,466	234.57	835.53	0.00	22,185.88
Mixed	1,726	268.16	733.74	0.00	4,313.33
True	3,593	323.19	867.40	0.00	18,273.06

Table S9: Summary statistics for the lifetime of conversations in conversations that had a fact checking comment.
KS-test for false and true conversations: $D = 0.139, p < 0.01$.

	N	Mean	SD	Min	Max
False	8,495	22.05	141.20	1	4,500
Mixed	2,874	25.56	140.81	1	3,437
True	6,704	21.91	121.97	1	4,407

Table S10: Summary statistics for the size of conversations in conversations without a fact checking comment.
KS-test for false and true conversations: $D = 0.021, p = 0.066$.

	N	Mean	SD	Min	Max
False	8,495	86.81	2049.70	1	183,762
Mixed	2,874	128.14	1470.07	1	65,830
True	6,704	61.31	385.76	1	12,443

Table S11: Summary statistics for the activity of conversations in conversations without a fact checking comment.
KS-test for false and true conversations: $D = 0.014, p = 0.434$.

	N	Mean	SD	Min	Max
False	8,495	4.22	5.74	1	196
Mixed	2,874	4.44	5.60	1	94
True	6,704	4.48	5.73	1	147

Table S12: Summary statistics for the maximum depth of conversations in conversations without a fact checking comment.
KS-test for false and true conversations: $D = 0.026, p = 0.013$.

	N	Mean	SD	Min	Max
False	8,495	70.10	473.03	0.00	19985.44
Mixed	2,874	66.35	337.68	0.00	9344.33
True	6,704	69.57	323.53	0.00	4288.05

Table S13: Summary statistics for the lifetime of conversations in conversations without a fact checking comment.
KS-test for false and true conversations: $D = 0.045, p < 0.01$.

	veracity	N	Mean	SD	Min	Max
1	false	5466	1769.72	6487.90	0.00	78358.00
2	mixed	1726	2939.61	8703.05	0.00	77732.00
3	true	3593	3509.94	10334.83	0.00	130682.00

Table S14: Summary statistics for the scores of posts that had received a fact-checking comment.
KS-test for false and true posts: $D = 0.146, p < 0.01$.

	veracity	N	Mean	SD	Min	Max
1	false	6596	26.57	174.73	-84.00	5943.00
2	mixed	2096	24.99	201.72	-109.00	6327.00
3	true	4052	41.54	368.14	-102.00	12133.00
4		40869				

Table S15: Summary statistics for the scores of fact-checking comments.
KS-test for false and true comments: $D = 0.053, p < 0.01$.
